# Supplementary material for: Exploring the influence of women’s leadership and corporate governance on operational liquidity: The glass cliff effect
Source: PLoS One. 2024 May 30;19(5):e0302210. doi: 10.1371/journal.pone.0302210 (PMC11139330; doi:10.1371/journal.pone.0302210)
Supplement: S1 Data — (PDF) [file pone.0302210.s001.pdf]

2018							
Company Name	WOB	BS	OBH	DQ	BD	CR	QR
1	0.00	8.00	0.93	0.63	0.50	1.12	0.07
2	0.00	8.00	0.87	0.38	0.88	1.60	1.40
3	0.00	7.00	0.46	0.57	0.57	0.23	0.04
4	0.00	9.00	0.56	0.33	0.56	2.92	1.60
5	0.14	7.00	0.73	0.14	0.57	1.36	0.05
6	0.10	10.00	0.58	1.00	0.80	1.22	0.21
7	0.00	7.00	0.12	0.43	1.00	5.64	4.08
8	0.10	10.00	0.46	0.40	0.40	1.77	0.63
9	0.14	7.00	0.54	0.71	0.57	1.34	0.64
10	0.00	7.00	0.84	0.29	0.86	0.87	0.13
11	0.00	8.00	0.22	0.75	1.00	1.72	0.06
12	0.14	7.00	0.31	0.57	1.00	1.29	0.74
13	0.14	7.00	0.31	0.57	1.00	1.29	0.74
14	0.00	10.00	0.39	0.40	0.40	1.49	0.40
15	0.00	9.00	0.51	0.56	0.44	4.84	1.14
16	0.14	7.00	0.79	0.71	0.57	0.71	0.13
17	0.22	9.00	0.00	0.33	0.78	2.12	1.21
18	0.00	8.00	0.55	0.25	1.00	1.36	0.43
19	0.13	8.00	0.47	0.50	0.50	1.36	0.43
20	0.14	7.00	0.00	1.00	0.86	0.11	0.01
21	0.00	7.00	0.67	0.71	0.86	1.45	0.85
22	0.33	9.00	0.37	0.11	0.11	1.14	0.45
23	0.13	8.00	0.75	0.63	0.25	1.80	0.88
24	0.00	7.00	0.63	0.14	0.71	1.11	0.38
25	0.00	10.00	0.69	0.80	1.00	0.89	0.36
26	0.14	7.00	0.32	0.86	0.71	1.02	0.11
27	0.00	7.00	0.11	0.86	0.43	1.44	0.34
28	0.11	9.00	0.00	0.33	0.56	2.01	0.62
29	0.00	9.00	0.00	0.67	0.89	1.16	0.12
30	0.00	11.00	0.56	0.36	0.91	1.13	0.05
31	0.00	7.00	0.70	0.43	1.00	1.12	0.55
32	0.10	10.00	0.37	0.80	0.90	1.15	0.45
33	0.13	8.00	0.38	0.50	0.75	0.89	0.12
34	0.00	12.00	0.50	0.58	0.83	0.89	0.12
35	0.08	13.00	0.45	0.85	1.00	0.96	0.64
36	0.14	7.00	0.56	0.71	0.43	1.31	0.37
37	0.00	9.00	0.32	0.44	0.78	1.09	0.35
38	0.00	9.00	0.56	0.56	0.89	1.42	0.83
39	0.00	7.00	0.15	1.00	0.57	1.32	0.87
40	0.18	11.00	0.76	0.64	0.82	0.75	0.12
41	0.08	12.00	0.32	0.75	0.92	0.95	0.26
42	0.00	11.00	0.53	0.55	0.64	0.85	0.25
43	0.11	9.00	0.49	0.67	0.56	1.14	0.41
44	0.00	8.00	0.10	0.25	0.88	2.21	0.57
45	0.13	8.00	0.00	0.50	0.63	0.86	0.39
46	0.00	10.00	0.30	0.50	0.80	1.08	0.28
47	0.29	7.00	0.78	0.71	0.86	1.96	1.03
48	0.14	7.00	0.83	0.57	0.57	2.09	0.87

49	0.25	8.00	0.25	0.25	0.38	5.14	2.14
50	0.14	7.00	0.72	0.57	0.71	4.83	2.30
51	0.00	7.00	0.57	0.57	0.57	2.11	0.60
52	0.07	14.00	0.29	0.57	0.79	1.02	0.85
53	0.00	13.00	0.91	0.69	0.85	0.95	0.57
54	0.14	7.00	0.36	0.29	0.57	1.45	1.23
55	0.00	7.00	0.54	0.71	0.86	1.29	1.15
56	0.00	7.00	0.51	0.57	0.57	2.18	1.67
57	0.00	10.00	0.00	0.40	0.90	1.08	0.79
58	0.00	7.00	0.15	0.29	0.86	1.24	0.38
59	0.00	9.00	0.68	0.78	0.78	0.79	0.37
60	0.13	8.00	0.25	0.75	0.63	0.34	0.10

2021							
**Company Name**	WOB	BS	OBH	DQ	BD	CR	QR
1	0.00	7.00	0.46	0.57	0.57	0.21	0.20
2	0.00	7.00	0.84	0.29	0.86	1.22	0.20
3	0.00	7.00	0.59	0.71	0.71	0.64	0.24
4	0.00	7.00	0.79	0.43	1.00	2.40	2.40
5	0.00	9.00	0.52	0.56	0.56	0.96	0.41
6	0.00	9.00	0.56	0.56	0.89	1.39	0.88
7	0.00	9.00	0.15	0.78	0.67	1.33	0.98
8	0.00	8.00	0.10	0.50	1.00	1.69	0.89
9	0.00	13.00	0.91	0.69	0.85	0.81	0.77
10	0.00	7.00	0.51	0.57	0.57	4.63	4.50
11	0.00	10.00	0.00	0.40	0.90	0.75	0.58
12	0.00	7.00	0.15	0.29	0.86	0.84	0.34
13	0.00	8.00	0.25	0.00	0.00	2.81	1.77
14	0.08	13.00	0.45	0.92	1.00	1.22	1.20
15	0.08	12.00	0.53	1.00	0.92	0.86	0.85
16	0.09	11.00	0.45	0.73	0.91	1.27	0.92
17	0.09	11.00	0.76	0.82	1.00	0.86	0.29
18	0.09	11.00	0.32	0.73	0.55	0.97	0.96
19	0.09	11.00	0.00	0.73	1.00	1.13	1.09
20	0.09	11.00	0.74	0.91	0.91	0.53	0.26
21	0.10	10.00	0.58	0.40	0.50	1.35	0.67
22	0.10	10.00	0.39	0.80	0.90	1.06	0.46
23	0.10	10.00	0.56	0.70	1.00	1.38	0.33
24	0.10	10.00	0.30	0.60	1.00	1.15	0.61
25	0.11	9.00	0.47	0.44	0.56	1.93	0.90
26	0.11	9.00	0.55	0.33	0.89	1.45	1.28
27	0.11	9.00	0.67	0.67	0.78	1.36	0.84
28	0.11	9.00	0.00	0.33	0.56	0.07	0.07
29	0.11	9.00	0.24	0.78	0.89	1.16	0.23
30	0.13	8.00	0.93	0.62	0.60	1.51	0.96
31	0.13	8.00	0.87	0.38	0.75	1.45	1.20
32	0.13	8.00	0.22	0.88	1.00	1.21	0.19
33	0.13	8.00	0.47	0.50	0.50	0.49	0.45
34	0.13	8.00	0.40	0.88	0.75	1.14	1.13
35	0.13	8.00	0.75	0.75	0.25	1.78	1.41
36	0.13	8.00	0.15	0.50	0.75	1.32	0.98
37	0.13	8.00	0.50	0.75	0.63	1.59	0.87
38	0.13	8.00	0.00	0.50	0.63	0.87	0.61
39	0.13	8.00	0.54	0.75	0.63	2.03	1.95
40	0.14	7.00	0.73	0.14	0.57	1.09	0.65
41	0.14	7.00	0.35	0.00	0.86	2.97	1.71
42	0.14	7.00	0.49	0.71	0.43	1.32	0.84
43	0.14	7.00	0.31	0.43	1.00	0.92	0.24
44	0.14	7.00	0.81	0.57	0.57	1.81	0.26
45	0.14	7.00	0.76	0.71	0.57	1.77	0.61
46	0.14	7.00	0.00	0.29	1.00	1.22	0.82
47	0.14	7.00	0.63	0.43	0.71	1.45	0.75
48	0.14	7.00	0.32	0.71	0.57	0.91	0.68

49	0.14	7.00	0.22	0.57	0.57	1.38	0.83
50	0.14	7.00	0.56	0.71	0.43	1.41	0.73
51	0.14	7.00	0.72	0.57	0.71	2.27	1.39
52	0.14	7.00	0.57	0.57	0.57	1.68	1.28
53	0.14	7.00	0.36	0.43	0.57	1.79	1.61
54	0.17	12.00	0.68	0.42	0.67	1.12	0.97
55	0.20	10.00	0.58	0.80	0.70	1.01	0.42
56	0.20	10.00	0.37	0.80	1.00	1.47	1.29
57	0.25	8.00	0.11	0.13	0.25	3.87	2.83
58	0.29	7.00	0.78	0.71	0.86	1.97	1.25
59	0.29	7.00	0.83	0.86	0.71	2.28	1.50
60	0.33	9.00	0.37	0.11	0.11	1.14	0.73
