# Supplementary material for: Exploring the influence of women’s leadership and corporate governance on operational liquidity: The glass cliff effect
Source: PLoS One. 2024 May 30;19(5):e0302210. doi: 10.1371/journal.pone.0302210 (PMC11139330; doi:10.1371/journal.pone.0302210)
Supplement: Attachment — Submitted filename: Respnse to Reviewers.docx [file pone.0302210.s002.docx]

| **Response to Reviewers** | | |
| --- | --- | --- |
| **Reviwer-1** | | |
|  | **Observations** | **Reply** |
| 1 | What motivated exploring the influence of women’s leadership and corporate governance on operational liquidity within the context of the Glass Cliff effect? Can you elaborate on why the Glass Cliff concept is particularly relevant to studying operational liquidity in the context of women’s leadership and corporate governance? | Several reasons led to the exploration of how corporate governance and women's leadership affect operational liquidity in the context of the Glass Cliff effect. Please see highlighted para 3 & 4 at Page No. 3 and Para 1 at Page. No. 4 |
| 2 | How specifically do the research objectives aim to address or contribute to our understanding of the Glass Cliff effect in the context of operational liquidity? | The aim of this research is to enhance to the body of knowledge already existing on the glass cliff effect---see para 1 at page No. 4 |
| 3 | Is there a specific theoretical framework that underpins the study, and how does it guide the exploration of the research objectives? | See para 3 and 4 at page No. 9 |
| 4 | The literature review provided in the article needs to establish a robust foundation to support the formulated hypothesis. While the introduction presents clear research objectives and questions related to the influence of women's leadership and corporate governance on operational liquidity, the literature review needs to adequately synthesize existing knowledge and evidence in a manner that logically leads to the proposed hypothesis. To enhance the quality of the literature review, it is recommended that the authors revisit and strengthen the synthesis of existing literature, emphasizing studies that directly contribute to the understanding of the Glass Cliff effect and its potential implications for operational liquidity. Also, the literature review should clearly show how the evidence being looked at led to the development of the hypothesis. This ensures a clear and logical flow from the existing body of knowledge to the specific research question being looked at. Addressing this gap will not only strengthen the study's theoretical foundation but also enhance its overall contribution to the field. | Literature review is revised as per instructions see page 5—8. |
| 5 | Some important relevant papers are missing in the literature review, for instance:  A. Exploring the China-Pakistan economic corridor project performance during Covid-19 pandemic.  B. Effect of firm structure on corporate cash holding (evidence from non-financial companies). | Relevant papers cited see at Page 3 & 4, also refences |
| 6 | The choice to utilize data spanning the years 2018 to 2021 is noted. However, considering the dynamic nature of business environments and the potential impact of external factors, it would be beneficial to understand the rationale behind not incorporating more recent data in the analysis. Recent years have witnessed significant global events, economic shifts, and changes in corporate landscapes that could influence the variables under investigation. Including more up-to-date information could provide a more accurate reflection of the current state of affairs and enhance the relevance and applicability of the study’s findings. The authors should address this concern by justifying the selected timeframe or, if feasible, incorporating more recent data to ensure the study’s results reflect the latest trends and developments in the field. | We employed data from 2018, prior to the onset of the COVID-19 pandemic, and data from 2021, during the pandemic, to examine change in configuration through the application of specialized software, fsQCA. The study utilized the fsQCA technique, involving a dialogue between theories and empirical data with a moderate number of cases, typically ranging between 15 and 65 (Berg-Schlosser et al., 2009). |
| 7 | The conclusion should go beyond summarizing the findings and reiterate the study's broader implications. It would be beneficial for the authors to contextualize the significance of their results within the existing literature, restate the critical contributions of the research, and discuss any unexpected or noteworthy findings. Additionally, the conclusion should tie back to the research objectives and emphasize how the study addresses the initial research questions.  While the current study makes a valuable contribution to understanding the influence of women's leadership and corporate governance on operational liquidity, it is essential to note that the article lacks a discussion on future research directions and policy implications. Addressing these aspects would significantly enhance the impact and practical relevance of the research. | Incorporated in discussion and conclusion, please see para 3 at page 18 and para 1 at page 19. |
| 8 | Consider incorporating a brief section that outlines potential avenues for future research. This could include suggestions for further exploring specific aspects of the Glass Cliff effect, operational liquidity, or related variables not covered in the current study. | Future research directions are incorporated and modified. See at page 19 |
|  | **Reviewer-2** | |
| 1 | •Can the author(s) enhance the motivation of their study even more?  •Additionally, the author(s) must present the sub-research questions and objectives in a concise and organized manner using bullet points. | Several reasons led to the exploration of how corporate governance and women's leadership affect operational liquidity in the context of the Glass Cliff effect. Please see highlighted para 3 & 4 at Page No. 3 and Para 1 at Page. No. 4 |
| 2 | The mere provision of the framework in section 2.6 by the author(s) is insufficient. Therefore, it is recommended that the author(s) include a detailed description of the proposed conceptual framework in the study. | Detailed description is added.  See para 3 and 4 at page No. 9 |
| 3 | •A proposed renaming of Section 3.0 to "Method" is desired.  •It is recommended that the authors substitute "relationship" for "connection."  •Figure 2 ought to be redrawn by the authors. | Needful done |
| 4 | The present discussion and concluding remarks fail to provide a clear depiction of the subject matter that the author intended to investigate. The following is the recommended order for the authors to rewrite this section:  1.The discussion section should be redrafted to explain extensively the implications of the findings. This should be done in line with previous studies.  2.Additionally, the implications of the study must be specified in detail.  3.The conclusion should also be redrafted ensuring that they restate the topic and its significance. In addition, the author(s) should restate the study's claim. Address opposing viewpoints and explain why the readers should support their viewpoint. Include a call to action or a summary of the possibilities and limitations of future research | Needful done in discussion and conclusion. See at Page 17 |
| **Reviewer-3** | | |
| 1 | I suggest to do more realistic analysis considering other corporate sectors too in other countries as well. Analysis outcome may differ. I suggest to enhance variable counts to be closer to realistic corporate environment | Please see at future directions of the study at Page 19. |
|  | | |
